# Comparison of *in vitro* antibiotic susceptibility testing of *R. typhi* using plaque assay and quantitative real-time PCR

**DOI:** 10.1128/spectrum.03595-25

**Published:** 2026-04-21

**Authors:** Weerawat Phuklia, Kaisone Padith, Christina M. Farris, Allen L. Richards, Koukeo Phommasone, Mayfong Mayxay, Mavuto Mukaka, Matthew T. Robinson, Paul N. Newton, Nicholas P. J. Day, Elizabeth A. Ashley

**Affiliations:** 1 Lao-Oxford-Mahosot Hospital-Wellcome Trust Research Unit, Microbiology Laboratory, Mahosot Hospital 251778, Vientiane, Laos; 2 Viral and Rickettsia Diseases Department, Infectious Disease Directorate, Naval Medical Research Center 19930, Silver Spring, Maryland, USA; 3 Allen L Richards PhD Consulting, Damascus, Maryland, USA; 4 Centre for Tropical Medicine and Global Health, Nuffield Department of Medicine, University of Oxford 105596, Oxford, United Kingdom; 5 Institute of Research and Education Development (IRED), University of Health Sciences, Ministry of Health 374369, Vientiane, Lao PDR; 6 Mahidol-Oxford Tropical Medicine Research Unit, Faculty of Tropical Medicine, Mahidol University 115374, Bangkok, Thailand; UCI Health, Orange, California, USA

**Keywords:** murine typhus, *Rickettsia typhi*, antibiotic susceptibility testing, MIC, plaque assay, qPCR

## Abstract

Murine typhus, caused by *Rickettsia typhi*, is a globally distributed flea-borne disease. Antimicrobial susceptibility data are largely based on reference strains, with little evaluation of variability across clinical isolates. The plaque assay, the gold standard for antibiotic susceptibility testing, requires up to 14 days to complete. Quantitative real-time PCR (qPCR) offers a rapid alternative for determining minimum inhibitory concentrations (MICs). Although qPCR-based susceptibility testing has been described, formal statistical assessment of agreement between methods remains limited. We estimated the MICs for azithromycin, doxycycline, and amoxicillin using qPCR and plaque assay on 24 *R. typhi* isolates (8 laboratory strains and 16 Lao clinical isolates) cultured in Vero cells. Heat-inactivated *R. typh*i (56°C, 30 min) served as a control. MIC was defined as the lowest concentration preventing plaque formation (plaque assay) or producing a Ct value greater than or equal to that of the heat-inactivated sample (qPCR). The mean MIC from three independent experiments was taken to represent each isolate. Agreement between methods was assessed using Bland-Altman analysis. Median qPCR MICs (MIC_50_) of all isolates were 0.130 mg/L (IQR, 0.104–0.240 mg/L) for azithromycin, 0.130 mg/L (IQR, 0.065–0.224 mg/L) for doxycycline, and 256 mg/L (IQR, 234.7–341.3 mg/L) for amoxicillin. Corresponding plaque assay MICs_50_ were 0.383 mg/L (IQR, 0.194–0.807 mg/L), 0.037 mg/L (IQR, 0.026–0.073 mg/L), and 170.7 mg/L (IQR, 112–256 mg/L), respectively. Bias was −0.3452 (−1.146, 0.4558) for azithromycin, 0.0969 (−0.2297, 0.4235) for doxycycline, and 85.33 (−187.3, 358) for amoxicillin. These findings demonstrate inter-isolate variability and quantitative agreement between methods.

**IMPORTANCE:**

Murine typhus is a treatable febrile illness caused by *Rickettsia typhi*, transmitted to humans via fleas. The plaque assay, which detects bacterial-induced host cell death in the presence or absence of antibiotics, is the reference method for testing drug susceptibility. However, the method requires approximately 2 weeks to obtain results. Detection of bacterial nucleic acid provides an alternative laboratory approach that reduces the assay duration to approximately 1 week, thereby improving research efficiency and throughput when testing multiple isolates. Compared with plaque assays, quantitative real-time PCR allows downstream analysis following sample inactivation, reducing prolonged high-containment handling. In this study, we compared the antibiotic susceptibility of 24 *R. typhi* isolates using both methods. We tested antibiotics commonly used to treat *R. typhi* infection (doxycycline and azithromycin), along with amoxicillin as a negative control. Both showed comparable results for all antibiotics tested, further supporting nucleic acid detection as a reliable and faster alternative.

## INTRODUCTION

Murine typhus is a neglected disease caused by the obligate intracellular bacterium, *Rickettsia typhi*, which is transmitted to humans via rat fleas (*Xenopsylla cheopis*) (1). The disease has a global distribution and, while usually treatable, it can lead to severe complications like meningoencephalitis (2). Doxycycline is the drug of choice for treating murine typhus (3). Azithromycin is an alternative drug given to pregnant women and young children. However, a clinical trial in Laos of azithromycin versus doxycycline in uncomplicated murine typhus observed increased numbers of clinical treatment failures in the azithromycin arm (4). One factor that could explain this finding is reduced drug susceptibility (5). However, antimicrobial susceptibility testing in obligate intracellular bacteria is very challenging compared to conventional methods for most extracellular bacteria.

The plaque assay is a method used to measure virus or *Rickettsia* titers by observing plaque formation following serial dilution of the pathogen. Plaques represent areas in a cell monolayer that are destroyed by the virus or bacteria during infection, indicating the presence of viable pathogens. In the presence of an antibiotic that inhibits pathogen replication, plaques do not form. This method has been applied to determine the minimum inhibitory concentrations (MICs) of antibiotics used for *Rickettsia* spp. and is considered the reference method for antibiotic susceptibility testing (6). However, this method is time-consuming due to the incubation period required for plaque formation, which varies depending on the *Rickettsia* species, at approximately 8–14 days (7).

Quantitative real-time PCR (qPCR), which detects bacterial DNA, has been used to determine MICs for obligate intracellular bacteria, including *Rickettsia* spp. (8). This method is faster than the plaque assay for some *Rickettsia* species, particularly *R. typhi* and *R. prowazekii* (7). MIC determination using qPCR is based on comparing bacterial DNA levels during infection with those observed in the presence of antibiotics, where a decrease indicates growth inhibition. Although several previous reports have documented that qPCR can be used to determine MIC in *Rickettsia* spp., it has mostly been tested on reference strains rather than on clinical isolates (8), and formal statistical evaluation of agreement between plaque assay and qPCR across multiple clinical isolates remains limited. Furthermore, the extent of inter-isolate variability in antimicrobial susceptibility among *R. typhi* strains has not been systematically assessed. Therefore, the objectives of this study were to evaluate antimicrobial susceptibility across multiple clinical and laboratory *R. typhi* isolates using established methods (qPCR and plaque assay) and quantitatively assess agreement between plaque assay and qPCR using Bland-Altman analysis.

## MATERIALS AND METHODS

### Host cell

African green monkey kidney cells (Vero; ATCC number CCL-81) were maintained in RPMI-1640 (Gibco, Invitrogen, USA) supplemented with 10% FBS (Sigma Aldrich, USA). The cells were incubated at 35°C in a humidified atmosphere with 5% CO_2_ overnight before the experiment, as described previously (9).

### *Rickettsia* strains and clinical isolates

Strains included in the evaluation were *R. typhi* strain Wilmington (obtained from the Australian Rickettsial Reference Laboratory, Geelong, Australia) and *R. typhi* strains AZ331, FLA6950, GER, GEAR, PAKNA, MUSSEIBOV, and TA837 (obtained from the Naval Medical Research Center, Bethesda, MD, USA) representing different regions of the world, as shown in Table 1. Additionally, 16 *R. typhi* clinical isolates were cultivated in Vero cells using buffy coat samples from Lao patients, as previously described (9, 10). These isolates were collected and stored as part of studies conducted by the Lao-Oxford-Mahosot Hospital-Wellcome Trust Research Unit (LOMWRU) in Vientiane, Lao PDR. These *Rickettsia* isolates are also listed in Table 1.

**TABLE 1:** List of *R. typhi* isolates and their geographic origin for antibiotic susceptibility testing

Isolate/strain	Geographic origin	Sample source	Year	Reference
AZ331	Ethiopia	*Rattus rattus*	1975-6	(11)
FLA H6590	Florida, USA	*Rattus rattus*	1951	(12)
GEAR	South Africa	Human	1939	(13)
GER	Republic of Georgia	Human	1946	(14)
MUSSEIBOV	Republic of Azerbaijan	Human	1949	(14)
NA18PP	Pakistan	Rat	1970	(11)
TA837	Thailand	*Rattus exulans*	1963	(11)
Wilmington	North Carolina, USA	Human	1928	(15)
TM1041	Vientiane Capital, Lao PDR	Human	2006	(9)
TM1377	Vientiane Capital, Lao PDR	Human	2007	(9)
TM2418	Vientiane Capital, Lao PDR	Human	2008	(9)
TM2504	Xayabury, Lao PDR	Human	2009	(9)
TM2522	Vientiane Capital, Lao PDR	Human	2009	(9)
TM2529	Vientiane Capital, Lao PDR	Human	2009	(9)
TM2540	Bolikhamxay, Lao PDR	Human	2009	(9)
TM2557	Vientiane Capital, Lao PDR	Human	2009	(9)
TM3627	Vientiane Capital, Lao PDR	Human	2010	(9)
TM3905	Vientiane Capital, Lao PDR	Human	2011	(9)
TM4034	Vientiane Capital, Lao PDR	Human	2011	(9)
TM4105	Khammouan, Lao PDR	Human	2012	(9)
TM4234	Vientiane Capital, Lao PDR	Human	2012	(9)
TM4237	Vientiane Capital, Lao PDR	Human	2012	(9)
TM8956	Vientiane Capital, Lao PDR	Human	2017	(9)
TM10184	Vientiane Capital, Lao PDR	Human	2019	(9)

### *Rickettsia* inoculum preparation

Before determining the MIC of antibiotics using qPCR and plaque assays, all *R. typhi* isolates were pre-grown at 35°C with 5% CO_2_ for 1 week from frozen stock ensuring the viability of the bacteria. The culture was then quantified by qPCR targeting the outer membrane protein B (*ompB*) gene, which is specific to *R. typhi* (16), to ensure that equal amounts of bacteria were used for inoculation. Briefly, Vero cells in a T25 flask were cultured at 35°C with 5% CO_2_ until they reached 80% confluence. The frozen *R. typhi* stock was rapidly thawed at 37°C in a water bath. The culture medium was discarded from the flask, and the suspension of infected cells was inoculated and incubated for 1.5 h at 35°C with 5% CO_2_. This incubation time allows bacteria to attach and enter the cells. After incubation, the inoculum was gently aspirated using a pipette, followed by two washes with 5 mL of 1× PBS. The Vero cells were resuspended in 5 mL of RPMI supplemented with 2% FBS and incubated at 35°C with 5% CO_2_ for 7 days. On Day 7, the old medium was discarded and replaced with 5 mL of fresh culture medium. The infected cells were detached using a cell scraper. A 1 mL aliquot of the infected cell suspension was transferred to a 1.5 mL microcentrifuge tube and vortexed at maximum speed to release *R. typhi* from the host cells. The suspension was centrifuged at 50 × *g* for 3 min to pellet cell debris. The supernatant was transferred to a new tube and spun at 20,238 × *g* for 5 min. qPCR was used to determine bacterial concentration in the resulting bacterial pellet.

To quantify the *R. typhi* DNA in the inoculum, DNA was extracted from the bacterial pellet using the HotShot method, as described previously (17). Briefly, 50 μL of alkaline lysis reagent was added to the microcentrifuge tube containing the bacterial pellet and mixed by pipetting. The mixture was heated at 95°C for 30 min, followed by the addition of 50 μL of neutralization reagent to stop the lysis reaction. A qPCR assay targeting the *ompB* gene was used to determine the bacterial load in the inoculum. Serial dilutions of plasmids containing the PCR target sequences, ranging from 10^0^ to 10^7^ copies/μL (pGEM-T kit; Promega, United Kingdom), were used as external controls for quantification (in duplicate).

Bacterial loads were estimated with the following formula: number of *R. typhi* DNA copies per mL of culture medium containing *R. typhi* pellet = (number of copies per PCR mixture using 1 μL DNA template) × 100. The factor of 100 accounts for the total 100 μL of extracted DNA from 1 mL of culture medium. The number of copies per reaction was determined using serial plasmid dilutions as external standards, resulting in the calculation of the number of copies per mL of culture medium (18).

### Antibiotic susceptibility testing for *R. typhi* isolates

#### MIC determination using qPCR

Vero cells (1 × 10^5^ cells/well) were plated in 24-well plates and incubated at 35°C with 5% CO_2_ until reaching 85%–90% confluent. The calculated *R. typhi* inoculum (adjusted to 1 × 10^6^ DNA copies per inoculum) was added to each well and incubated for 1.5 h at 35°C with 5% CO_2_. After incubation, the inoculum was gently and completely aspirated using a micropipette to avoid disturbing the cell monolayer, and the cells in each well were washed with 1 mL 1× PBS. RPMI media with and without serial dilutions of azithromycin (ranging from 0.0039 mg/L to 2 mg/L), doxycycline (ranging from 0.0039 mg/L to 1 mg/L), and amoxicillin (ranging from 1 mg/L to 512 mg/L) were added. These antibiotics were freshly prepared for each MIC experiment from antibiotic stock solutions. Briefly, azithromycin and doxycycline were reconstituted in dimethyl sulfoxide at 10 mg/mL to prepare stock solutions, whereas amoxicillin was reconstituted in sterile distilled water at 10 mg/mL. All stock solutions were sterilized by filtration through 0.22 μM syringe filters, aliquoted into 0.2 mL volumes, stored at −20°C, and thawed only once prior to use. The plates were incubated for 7 days at 35°C with 5% CO_2_ before MIC determination. *R. typhi* without antibiotic at Day 0 served as a control to confirm the initial bacterial input in each assay. *R. typhi* without antibiotic at Day 7 served as the positive growth control. Heat-inactivated *R. typhi* (56°C for 30 min) served as the negative control and baseline reference for MIC determination (19).

After 7 days, infected cells were harvested, centrifuged, and DNA was extracted using the HotShot method (17). Bacterial DNA concentration was determined by Taqman-qPCR targeting the *ompB* gene (16). The MIC by qPCR was defined as the antibiotic concentration corresponding to a Ct value equal to or higher than that of the heat-inactivated sample.

#### MIC determination using plaque assay

Vero cells were plated in 24-well plates using the same condition as those used for MIC determined by qPCR. *R. typhi* inoculum (1 × 10^6^ copies per inoculum) was then added to the monolayer. After 1.5 h of incubation, an overlay medium (2× RPMI with 8% FBS mixed with 1% agarose gel at a ratio of 1:1) containing serial dilutions of azithromycin (0.0039–2 mg/L), doxycycline (0.0039–1 mg/L), and amoxicillin (1–512 mg/L), or without antibiotics, was added. The plates were then incubated for 14 days to allow *R. typhi* plaque formation. On Day 12, 0.01% neutral red in PBS was added to each well and incubated at 35°C with 5% CO_2_ overnight, protected from light. The staining solution was removed the following day. The plaque assay procedure was adapted from the *Rickettsia rickettsii* quantification protocol, as previously described (19). On Day 14, plaque formation was observed in cultures with and without antibiotics. The MIC was determined as the lowest antibiotic concentration that inhibited plaque formation.

### Statistical analysis

The average MIC for each isolate, calculated from three independent experiments, was used as the representative MIC (Data set S1, Figshare, https://doi.org/10.6084/m9.figshare.31274773). The normality of the 24 isolate-level MIC values was assessed using the Shapiro-Wilk test. As MIC data were not normally distributed in the majority of groups (*P* < 0.05), a non-parametric test was used for MIC comparison between methods.

The median (IQR) MICs for each *R. typhi* isolate from the two methods have been reported, and the distributions of the paired MIC values were compared using the Wilcoxon signed-rank test. To test whether MICs determined by qPCR and plaque assay were comparable, agreement between the two methods was assessed using the Bland-Altman method (20). Briefly, the mean difference in MIC values obtained from qPCR and plaque assay was calculated. To evaluate whether the difference between the two methods was acceptable, the 95% limits of agreement (LoA) were used. This approach quantifies agreement between two measurements by studying the mean difference and constructing LoA (21). The Bland-Altman plots were obtained, and tests of significance have been performed at 5% significance level. Statistical analyses were conducted using GraphPad Prism version 10.6.1 (GraphPad Software, Inc., San Diego, CA, USA).

## RESULTS

### Determination of the MICs of azithromycin, doxycycline, and amoxicillin against 24 *R. typhi* isolates using qPCR and plaque assay

The median MIC values (MIC_50_) for azithromycin, doxycycline, and amoxicillin against the 24 *R. typhi* isolates determined by qPCR were 0.130 mg/L (IQR, 0.104–0.240 mg/L), 0.130 mg/L (IQR, 0.065–0.224 mg/L), and 256 mg/L (IQR, 234.7–341.3 mg/L), respectively. Similarly, the median MIC values determined by plaque assay were 0.383 mg/L (IQR, 0.194–0.807 mg/L) for azithromycin, 0.037 mg/L (IQR, 0.026–0.073 mg/L) for doxycycline, and 170.7 mg/L (IQR, 112–256 mg/L) for amoxicillin, as listed in Table 2.

**TABLE 2:** The representative mean MICs from three independent experiments for azithromycin, doxycycline, and amoxicillin, as determined by qPCR and plaque assay, for each *R. typhi* isolate, along with the MIC_50_ and MIC_90_ values of the three antibiotics against 24 *R. typhi* isolates^*a*^

Isolate	Azithromycin (mg/L)		Doxycycline (mg/L)		Amoxicillin (mg/L)	
	qPCR	Plaque assay	qPCR	Plaque assay	qPCR	Plaque assay
TM8956	0.115	1.354	0.036	0.034	362.667	64.000
TM1041	0.250	1.167	0.208	0.021	256.000	106.667
TM2557	0.250	0.583	0.083	0.344	426.667	512.000
TM2418	0.125	0.833	0.146	0.096	234.667	64.000
TM3905	0.250	0.729	0.375	0.135	170.667	256.000
TM2522	0.250	0.677	0.125	0.188	213.333	170.667
TM1377	0.073	0.094	0.063	0.042	256.000	362.667
TM2540	0.104	1.000	0.229	0.229	256.000	341.333
TM2504	0.167	1.167	0.229	0.073	341.333	128.000
NA 18PP	0.208	0.089	0.063	0.023	277.333	149.333
GER	0.167	0.182	0.146	0.026	256.000	192.000
TA837	0.073	1.005	0.135	0.026	149.333	170.667
GEAR	0.125	0.354	0.688	0.026	362.667	64.000
MUSEIBOV	0.250	0.375	0.250	0.036	426.667	256.000
TM4034	0.333	0.052	0.208	0.026	384.000	128.000
TM4105	0.125	0.385	0.083	0.036	234.667	64.000
TM4234	0.073	0.229	0.052	0.034	85.333	256.000
TM4237	0.104	0.380	0.125	0.063	341.333	170.667
TM3627	0.104	0.521	0.052	0.036	234.667	213.333
FLA H6590	0.208	0.229	0.083	0.026	341.333	213.333
AZ331	0.208	0.057	0.146	0.031	341.333	149.333
Wilmington	0.107	0.292	0.073	0.073	85.333	213.333
TM10184	0.104	0.052	0.375	0.052	341.333	256.000
TM2529	0.135	0.385	0.052	0.023	256.000	85.333
Median (MIC_50_)	0.130	0.383	0.130	0.036	256.0	170.7
IQR	0.104–0.240	0.194–0.807	0.065–0.224	0.026–0.073	234.7–341.3	112–256
MIC_90_	0.250	1.118	0.338	0.172	377.6	315.7
MIC range	0.073–0.333	0.052–1.354	0.037–0.688	0.021–0.323	85.33–426.7	64–512
Coefficient of variation (%)	44.68	78.14	86.99	111.7	34.09	56.58
Skewness	0.624	0.716	2.248	2.396	−0.399	1.198
Kurtosis	−0.600	−0.620	6.289	5.803	−0.264	2.061
Wilcoxon matched-pairs signed rank test	*P* = 0.0002		*P* = 0.0010		*P* = 0.0069	

^*a*^ *P* < 0.05 cutoff was used for statistical significance.

**Fig 1:**
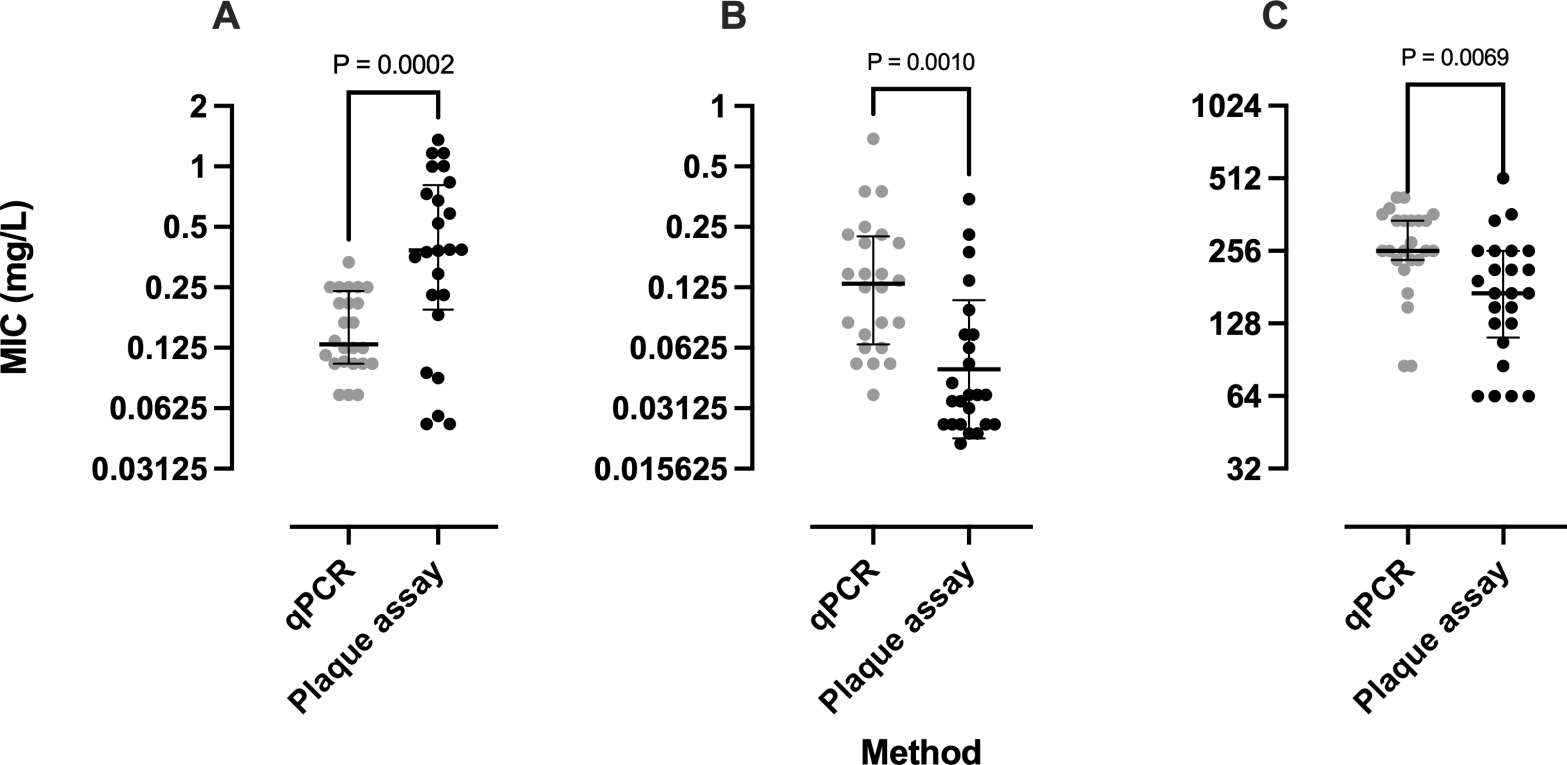
The scatter plots represent the median MIC determined by qPCR and plaque assay for three antibiotics: azithromycin (**A**), doxycycline (**B**), and amoxicillin (**C**) against 24 *R. typhi* isolates. The gray circles represent the MIC based on qPCR, and the black circles represent the MIC based on plaque assay. *P* value <0.05 indicates statistically significant differences in MIC values, as determined by the Wilcoxon test, when comparing qPCR and plaque assay results.

The distribution of the MIC values determined by qPCR and plaque assay was compared for each antibiotic. A threefold difference in median MIC values was observed between the methods for azithromycin, with the MIC value determined by qPCR being lower than that by plaque assay. However, approximately fourfold and twofold differences in median MIC values were observed for doxycycline and amoxicillin, respectively, with MIC values determined by qPCR being higher than those by plaque assay, as shown in Fig. 1.

### Analysis of agreement and systematic bias

**Fig 2:**
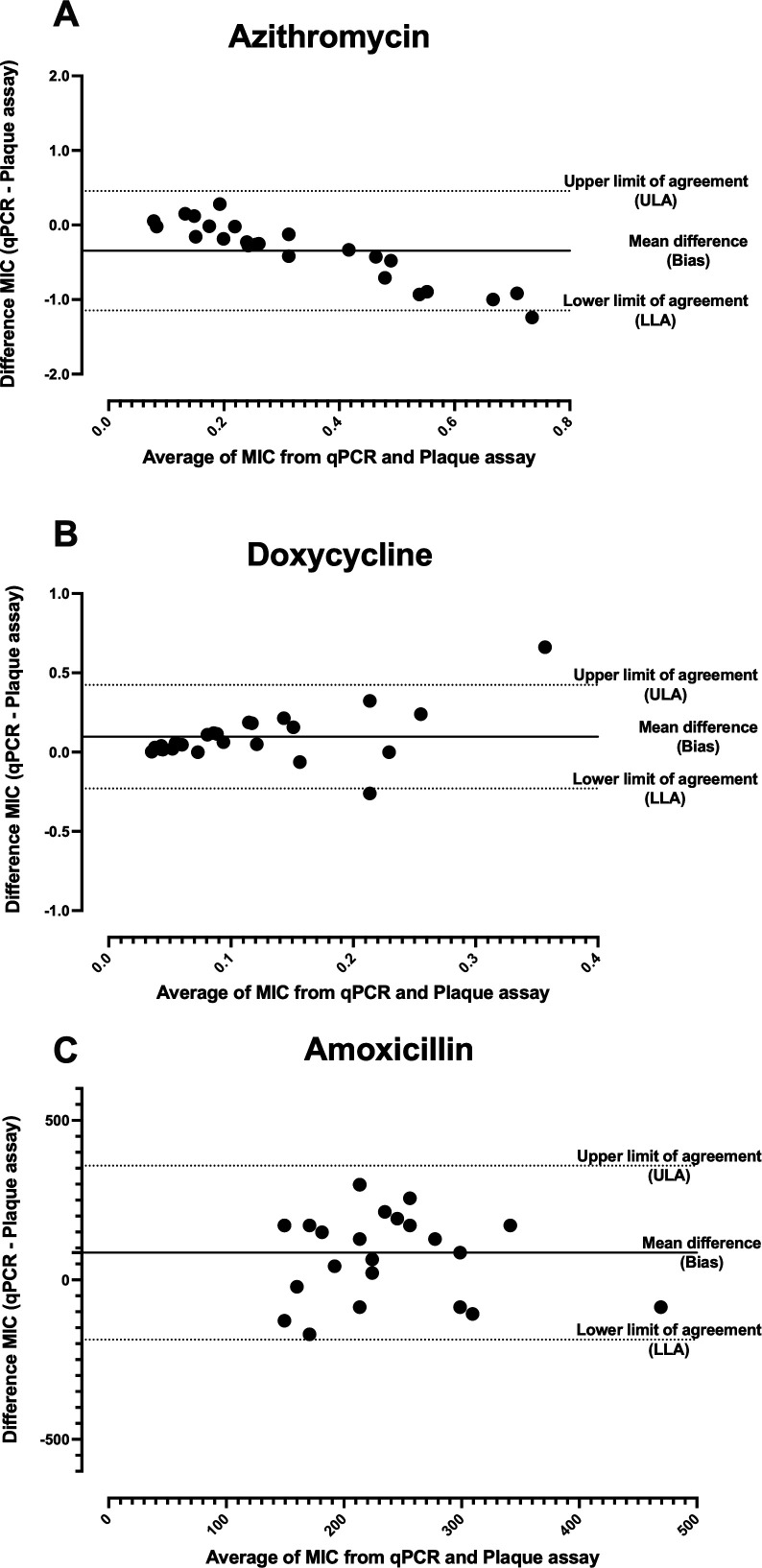
Bland-Altman difference plots comparing the MIC results of the qPCR method and plaque assay for azithromycin (A), doxycycline (B), and amoxicillin (C) are shown. The mean difference between the methods was used as the bias. The 95% limits of agreement, upper limit of agreement and lower limit of agreement, were used to define the LoA. The *x*-axis represents the average MIC between two methods, and the *y*-axis represents the difference in MIC between two methods. The plots confirm that measurement by both methods was comparable for all three antibiotics.

Bland-Altman difference plots were used to assess the limit of agreement and bias between the two methods, as shown in Fig. 2.

The mean difference between methods (qPCR and plaque assay) and the limits of agreement (lower limit and upper limit) for MIC determination of azithromycin, doxycycline, and amoxicillin are listed in Table 3. For azithromycin, the bias of MIC (or mean difference) between qPCR and plaque assay was −0.3452, indicating that the MIC determined by qPCR was 0.3452 units lower than that determined by plaque assay. The bias fell within the limits of agreement (−1.146 and 0.456). For doxycycline, the bias was 0.0969, meaning that the MIC measured by qPCR was 0.0969 units higher than that measured by plaque assay, and it fell within the limits of agreement (−0.230 to 0.424). In the case of the amoxicillin group, the bias was 85.33 mg/L, and the value was within the range of limits of agreement (−187.3 and 358 mg/L), indicating that the MIC measured by qPCR was 85.33 units higher than that determined by plaque assay.

**TABLE 3:** Bland-Altman analysis for antibiotic susceptibility testing methods using qPCR and plaque assay for azithromycin, doxycycline, and azithromycin

Bland-Altman analysis	Azithromycin	Doxycycline	Amoxicillin
Bias	−0.3452	0.0969	85.33
SD of bias (standard deviation of difference)	0.4087	0.1667	139.1
95% LoA			
Lower limit of agreement	−1.146	−0.230	−187.3
Upper limit of agreement	0.456	0.424	358

## DISCUSSION

In this study, we determined the MICs of doxycycline, azithromycin, and amoxicillin for 24 *R. typhi* isolates using two methods. To validate the qPCR and plaque assay methods, this study aimed to evaluate whether MIC determination using qPCR is comparable to the gold standard method, the plaque assay (6). Although antimicrobial susceptibility testing methods for *rickettsiae* are not routinely performed in clinical laboratories, as they are time-consuming and there are no established breakpoints to define susceptibility or resistance, the plaque assay remains, within experimental rickettsial research, the only established viability-based method that directly measures infectious, replication-competent *Rickettsia* through host cell infection and lysis in the presence of antibiotics. For this reason, it has been consistently used as the gold standard in previous studies evaluating alternative approaches, including qPCR-based MIC determination (8). In this experimental context, the plaque assay has functioned as the reference method (6) against which qPCR is evaluated. As expected, the amount of DNA detected during drug testing was higher than the number of viable *Rickettsia* determined by the plaque assay. However, the MIC of the antibiotic was the parameter used for comparison, rather than the amount of DNA or viable bacteria.

Previous studies comparing MIC determination using the plaque assay and qPCR for *Rickettsia* spp. including *R. typhi* have been reported (8, 22). However, these studies used only reference strains rather than isolates from different geographic origins or patient-derived isolates, which may not represent the full variation in drug susceptibility among isolates. In this study, the MICs of azithromycin, doxycycline, and amoxicillin were measured using both qPCR and plaque assay methods. We performed three independent experiments to test for reproducibility. Although overall agreement between methods was observed, variations were noted for certain isolates, with some exhibiting larger inter-assay MIC differences. These discrepancies may be attributable to methodological and experimental factors rather than intrinsic resistance. Differences in host cell passage number may have influenced bacterial replication dynamics and, consequently, MIC determination in both assays. While the same inoculation stock was used whenever possible, variations in handling time and storage conditions prior to infection may have affected bacterial viability and subsequent growth. In addition, differences in the preparation, handling, and storage of antibiotic stock solutions between experiments may have contributed to MIC variability.

Fresh medium containing antibiotics was not replenished during incubation; therefore, potential antibiotic degradation over time may have influenced MIC measurements. This effect may have been more pronounced in the plaque assay, in which MICs were determined after approximately 14 days of incubation compared with 7 days for the qPCR-based method. The longer incubation period and distinct biological endpoints of the plaque assay may differentially capture antibiotic effects, potentially contributing to the larger MIC differences observed for some isolates.

The median MICs of doxycycline and amoxicillin determined by qPCR were higher than those determined by plaque assay, indicating that qPCR might detect both dead and live bacterial cells, whereas plaque assay detects only viable *Rickettsia*. However, the median MIC of azithromycin determined by qPCR was lower than that determined by plaque assay, in contrast to doxycycline and amoxicillin.

This finding may be explained by azithromycin’s intracellular accumulation and lysosomal trapping in both phagocytic and non-phagocytic cells (23). Vero cells, derived from African green monkey kidney epithelium (24), are widely used as a non-phagocytic epithelial model for study *Rickettsia* spp. (25, 26). Like other epithelial cells, Vero cells contain acidic endosomal and lysosomal compartments that sequester weakly basic drugs such as azithromycin (23), and *R. typhi* replicates freely in the host cytosol (27). If its release from these compartments is slow, low azithromycin levels, which inhibit bacterial protein synthesis (28), may suppress bacterial DNA replication earlier than they block host cell infectivity and spread.

In this study, we assessed agreement between methods using the Bland-Altman method. The mean difference (bias) for MIC determination using qPCR was slightly different from that using the plaque assay for azithromycin and doxycycline but remained within the clinically acceptable range of one twofold dilution (29), suggesting that MIC determination by qPCR is comparable to the plaque assay for these antibiotics. Although the mean difference in MICs between qPCR and plaque assay was close to zero for both azithromycin and doxycycline, the direction of the difference was inconsistent.

The bias between methods for amoxicillin fell within the acceptance limit; the value was not close to zero (85.33 mg/L), indicating that the two methods are comparable but not identical. This might be because both methods consistently indicate poor amoxicillin activity; indeed, this antibiotic is not clinically recommended against *Rickettsia* spp. (22).

This study has three main limitations. First, the number of *R. typhi* isolates was small. Only a few research groups worldwide study and isolate this pathogen, limiting the availability of isolates from different geographical regions. Second, doxycycline-resistant and azithromycin-resistant *R. typhi* strains have not been described. Such strains would be necessary to standardize current antibiotic susceptibility testing methods and to determine their ability to distinguish between resistant and susceptible isolates. Third, antibiotic replenishment was not performed for either method, and antibiotic degradation at 35°C during prolonged incubation is possible and may have influenced the absolute MIC values. Consequently, validated susceptibility or resistance cutoff values are not currently available for *R. typhi* using either method, and MIC results should therefore be interpreted for comparative purposes only.

Investigating diverse *R. typhi* isolates from additional regions will provide valuable MIC profile data for both methods and may contribute to establishing MIC breakpoints. Additionally, developing a high-throughput antibiotic susceptibility assay will enable testing of multiple drugs and isolates simultaneously, by scaling from a 24-well to a 96-well plate format. Using a 96-well plate will allow optimization of MIC determination with fresh blood-derived bacteria, rather than high passage cultures, to obtain more accurate MIC values. These advancements will facilitate drug screening assays for both existing and novel treatments for rickettsial infections and improve our understanding of antibiotic susceptibility, including the establishment of breakpoints for clinical *R. typhi* isolates.

### Conclusion

Antibiotic susceptibility testing for *Rickettsia typhi* isolates using qPCR is comparable to the gold standard method, the plaque assay, for testing susceptibility to doxycycline and azithromycin. Using qPCR to determine the MIC of antibiotics for *Rickettsia typhi* reduces the time to obtain results by a week compared to the plaque assay.

## Data Availability

The data supporting the findings of this study are available within the article. Additional data are available in Data set S1 deposited in the Figshare repository (https://doi.org/10.6084/m9.figshare.31274773).
